# High-Dose-Rate Brachytherapy Monotherapy versus Image-Guided Intensity-Modulated Radiotherapy with Helical Tomotherapy for Patients with Localized Prostate Cancer

**DOI:** 10.3390/cancers10090322

**Published:** 2018-09-10

**Authors:** Hideya Yamazaki, Koji Masui, Gen Suzuki, Satoaki Nakamura, Daisuke Shimizu, Tatsuyuki Nishikawa, Haruumi Okabe, Ken Yoshida, Tadayuki Kotsuma, Eiichi Tanaka, Keisuke Otani, Yasuo Yoshioka, Kazuhiko Ogawa

**Affiliations:** 1 Department of Radiology, Graduate School of Medical Science, Kyoto Prefectural University of Medicine, 465 Kajiicho Kawaramachi Hirokoji, Kamigyo-ku, Kyoto 602-8566, Japan; mc0515kj@koto.kpu-m.ac.jp (K.M.); gensuzu@koto.kpu-m.ac.jp (G.S.); satoaki@nakamura.pro (S.N.); dshimizu@koto.kpu-m.ac.jp (D.S.); 2 Department of Radiology, Ujitakeda Hospital, Uji-City, Kyoto 611-0021, Japan; nishikawa2017@excite.co.jp (T.N.); h-okabe@takedahp.or.jp (H.O.); 3 Department of Radiation Oncology, National Hospital Organization Osaka National Hospital, 2-1-14, Hoenzaka, Chuo-ku, Osaka 540-0006, Japan; rad113@osaka-med.ac.jp (K.Y.); tkotsuma-osaka@umin.net (T.K.); tanaka@onh.go.jp (E.T.); 4 Department of Radiation Oncology, Osaka University Graduate School of Medicine, Suita, Osaka 565-0871, Japan; ohtanik@radonc.med.osaka-u.ac.jp (K.O.); yasuo.yoshioka@jfcr.or.jp (Y.Y.); kogawa@radonc.med.osaka-u.ac.jp (K.O.)

**Keywords:** prostate cancer, high-dose-rate brachytherapy, image guided intensity modulated radiotherapy

## Abstract

The aim of this paper is to compare outcomes between high-dose-rate interstitial brachytherapy (HDR-BT) monotherapy and image-guided intensity-modulated radiotherapy (IG-IMRT) for localized prostate cancer. We examined 353 HDR-BT and 270 IG-IMRT patients. To reduce background selection bias, we used the method of inverse probability treatment weighting (IPTW) with propensity scores. The actuarial five-year biochemical failure-free survival rates were 92.9% and 96.7% (*p* = 0.1847; *p* = 0.077 in IPTW) for HDR-BT and IG-IMRT, respectively; they were 100% and 95.8% (*p* = 0.286) for the low-risk group, 95.6% and 92% (*p* = 0.42) for the intermediate-risk group, 90.4% and 84.9% (*p* = 0.1059; *p* = 0.04 in IPTW) for the high-risk group, and 87.1% and 89.2% (*p* = 0.3816) for the very-high-risk group. In the assessment of accumulated incidences of grade ≥ 2 toxicity (Common Terminology Criteria for Adverse Events version 4.0) at five years, HDR-BT monotherapy showed higher genitourinary toxicity (11.9%) than IG-IMRT (3.3%) (*p* < 0.0001). The gastrointestinal toxicity was equivalent for HDR-BT (2.3%) and IG-IMRT (5.5%) (*p* = 0.063). No Grade 4 or 5 toxicity was detected in either modality. HDR-BT showed higher genitourinary toxicity than IG-IMRT. HDR-BT and IG-IMRT showed equivalent outcomes in low-, intermediate-, and very-high-risk groups. For high-risk patients, HDR-BT showed potential to improve prostate-specific antigen (PSA) control rate compared to IG-IMRT.

## 1. Introduction

Prostate cancer is one of the most prevalent noncutaneous malignancies among men in Western countries, and it has been increasing in incidence in recent decades. The main reasons for this prevalence are increasing life expectancy, marked presence of the Western lifestyle (high-calorie diet and sedentary lifestyle), and improvement in accurate diagnostic methods. Currently, common treatment options include prostatectomy, external beam radiotherapy (EBRT), and interstitial brachytherapy (BT) [1].

With the advancement of EBRT techniques, image-guided intensity-modulated radiotherapy (IG-IMRT) has become more widely used for prostate cancer. IG-IMRT can reduce normal tissue toxicity compared to three-dimensional conformal radiotherapy (3D-CRT) or even IMRT [2,3,4]. As a result, advanced EBRT is now a standard treatment for all stages of localized prostate cancer [1,4]. In our earlier studies, we used IG-IMRT with helical tomotherapy—with or without hormonal therapy—which enabled precise dose delivery using megavoltage-computed tomography (MVCT) [5,6].

BT can deliver a high dose of radiation to the prostate gland by avoiding surrounding normal tissue. It is therefore regarded as an effective radiotherapy treatment option [7] that could improve outcomes with long-term biochemical control in two ways: (i) permanent implantation and low-dose-rate (LDR) BT or (ii) temporary implantation and high-dose-rate (HDR) BT. HDR-BT monotherapy is an established treatment option for patients with low- to intermediate-risk prostate cancer with excellent long-term outcome data [1]. HDR has also been employed as a booster technique delivered concurrently with EBRT (HDR-BT plus EBRT) for patients with intermediate- and high-risk prostate cancer [1,7]. On the other hand, several authors have used HDR-BT as a monotherapy and reported excellent outcomes for all risk groups [8,9,10,11]. If this could be confirmed, it would be the most efficient method of achieving good dose distribution with a high degree of conformity—even for adjacent tissue invasion (seminal vesicle or extracapsular extension)—and a short overall treatment time.

To date, several trials—including randomized controlled trials (RCTs)—have demonstrated benefits of biochemical control through dose escalation [1,4,11,12,13]. Although three RCTs (EBRT vs. EBRT + BT boost) have compared the treatment effectiveness of those RT modalities [11,12,13], there have been few direct comparisons between modern IG-IMRT using helical tomotherapy and HDR-BT monotherapy. For this reason, the efficacy of HDR-BT in relation to that of IG-IMRT has not yet been established. The interpretation of retrospective evidence can be challenging, partly because of the extensive background differences. Therefore, in this study, we introduced the method of inverse probability of treatment weighting (IPTW) using propensity scores to reduce background selection bias. We compared the outcomes of patients treated with IG-IMRT with helical tomotherapy to those of patients treated with HDR-BT monotherapy to determine the rationale for HDR-BT based on current clinical outcomes.

## 2. Results

### 2.1. Patient Characteristics

The median follow-up for the entire cohort was 76 months (range: 12–216), with a minimum of one year for surviving patients or until death. A comparison of the backgrounds of the two modalities is given in Table 1. HDR-BT monotherapy-treated patients had lower pretreatment prostate-specific antigen (PSA) levels, lower Gleason scores (GSs) with more hormonal therapy, and longer follow-up periods compared to patients treated with IG-IMRT tomotherapy.

### 2.2. Biochemical Control and Survival

**Figure 1:**
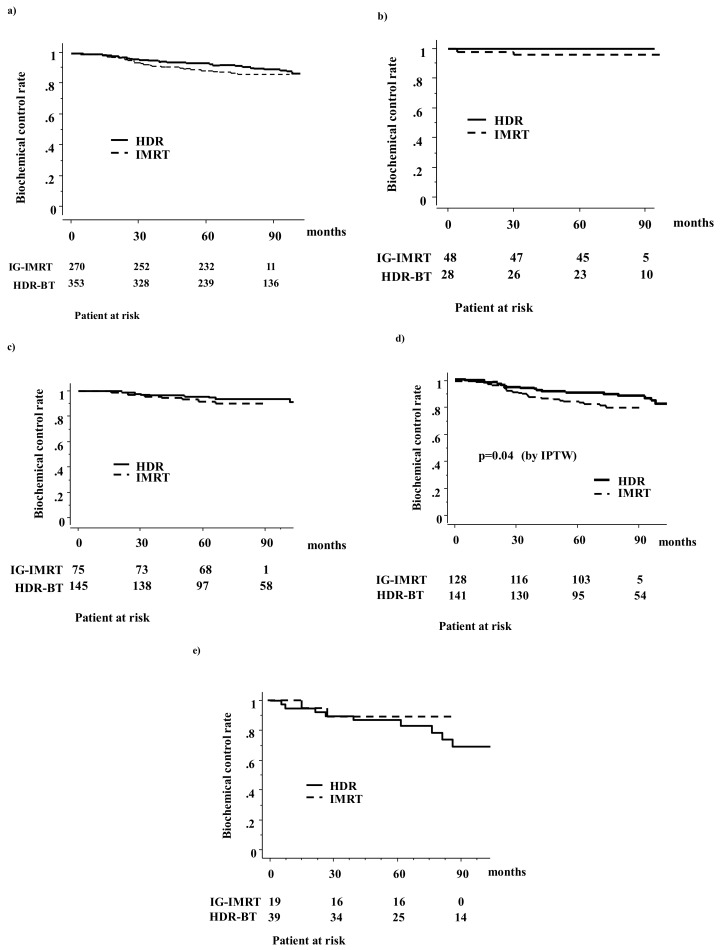
Biochemical control rates between HDR-BT monotherapy and IG-IMRT with helical tomotherapy: (**a**) total population; (**b**) low-risk group; (**c**) intermediate-risk group; (**d**) high-risk group; (**e**) very-high-risk group.

In the HDR-BT group, 39 (10.9%) patients developed biochemical failure, whereas 34 (12.6%) did so in the IG-IMRT group (Figure 1). The actuarial five-year biochemical failure-free survival rate was 92.9% (95% confidential interval (CI) = 90.1–95.6%) and 89.2% (95% CI = 85.9–92.9%, *p* = 0.18; *p* = 0.077 in IPTW) for HDR-BT and IG-IMRT, respectively; they were 97.3% (100% for HDR-BT and 95.8% for IG-IMRT, *p* = 0.28; *p* = 0.15 in IPTW) for the low-risk group, 94.2% (95.6% for HDR-BT and 92% for IG-IMRT, *p* = 0.42; *p* = 0.60 in IPTW) for the intermediate-risk group, 87.7% (90.4% for HDR-BT and 84.9% for IG-IMRT, *p* = 0.10; *p* = 0.041 in IPTW) for the high-risk group, and 87.8% (87.1% for HDR-BT and 89.2% for IG-IMRT, *p* = 0.38; *p* = 0.60 in IPTW) for the very-high-risk group. There was a significant difference in the biochemical control rate among the four risk groups (*p* = 0.0004). Thus, a statistically significant difference was found for the high-risk group in IPTW (Figure 1 and Table 2).

As shown in Table 3, T classification, pretreatment PSA levels, and National Comprehensive Cancer Network (NCCN) risk classification were the predictors of biochemical control on univariate analysis. In multivariate Cox regression analysis, the T category and pretreatment PSA levels remained significant indicators for improving biochemical control.

As there were only three prostate cancer-related deaths (one very-high-risk and two high-risk patients who underwent HDR-BT died of prostate cancer at 55, 75, and 157 months after treatment, respectively), the five-year cause-specific survival rates were 99.8% (99.6% for HDR-BT and 100% for IG-IMRT, *p* = 0.17) for all groups.

The overall five-year survival rates were 97.4% (95% CI = 95.6–99.2%) and 99.6% (95% CI = 98.9–100.4%, *p* = 0.96) for HDR-BT and IG-IMRT (HR = 1.105, 95% CI = 0.534–2.286, *p* = 0.7874), respectively; they were 100% (100% for HDR-BT and 100% for IG-IMRT, *p* = 0.66) for the low-risk group, 99.0% (98.4% for HDR-BT and 100% for IG-IMRT, *p* = 0.44) for the intermediate-risk group, 96.9% (94.7% for HDR-BT and 99.2% for IG-IMRT, *p* = 0.98) for the high-risk group, and 97.9% (96.7% for HDR-BT and 100% for IG-IMRT, *p* = 0.49) for very-high-risk group. There were no statistically significant differences among the four risk groups (*p* = 0.89) in their overall survival rates.

### 2.3. Late Toxicity

Late GI toxicities of Grades 1, 2, and 3 occurred in 33 (9%), 10 (3%), and 1 (0.3%) patients on HDR-BT and in 37 (9%), 10 (3%), and zero (0%) on IG-IMRT, respectively (*p* = 0.094) (Table 4). Late GU toxicities of Grades 1, 2, and 3 occurred in 100 (28%), 57 (16%), and 10 (3%) patients on HDR-BT and in 36 (13%), 11 (4%), and 1 (0.3%) patients on IG-IMRT (*p* < 0.0001), respectively. HDR-BT resulted in more GU toxicity (*p* < 0.0001) and equivalent GI toxicity (*p* = 0.094) compared to IG-IMRT. No complications of a grade higher than or equal to Grade 4 were observed in either arm.

**Figure 2:**
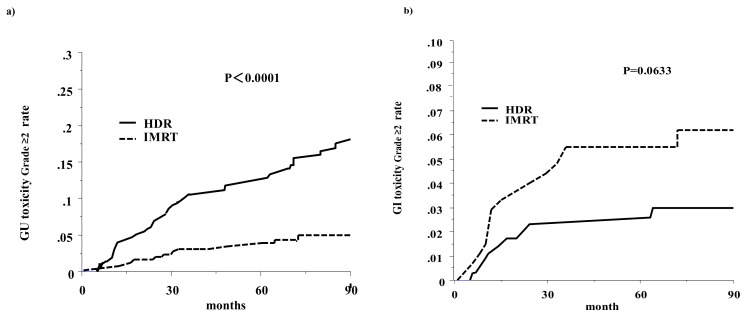
The accumulated incidence of grade ≥ 2 toxicity: (**a**) Genitourinary toxicity; (**b**) Gastrointestinal toxicity.

The accumulated incidence of grade ≥ 2 toxicity is shown in Figure 2. At five years, the accumulated incidences of grade ≥ 2 GI toxicity were 2.3% and 5.5% for HDR-BT and IG-IMRT (*p* = 0.063), respectively. For GU toxicity, it was 11.9% and 3.3% for HDR-BT and IG-IMRT (*p* < 0.0001), respectively. The HDR-BT showed higher grade ≥ 2 GU toxicity than the IG-IMRT. Multivariate analyses also revealed that the HDR-BT schedule was the only statistically significant predictive factor for grade ≥ 2 GU toxicity with a hazard ratio of 3.91 (95% confidential interval: 2.07–7.39, *p* < 0.0001; Table 5).

For GI toxicity, five patients showed rectal bleeding grade 3, 6–36 months later (median 12 months) after treatment with IG-IMRT. In addition, one patient treated with HDR-BT showed a urethrorectal fistula.

For Grade 3 GU toxicity, one hematuria appeared in the IG-IMRT arm (16 months), whereas 10 HDR-BT patients showed three hematuria and seven obstructions

## 3. Discussion

A recent study of multiple RCTs had demonstrated that an increase in the prescribed dosages improved biochemical control [1,4]. Taking this result as a given, we considered that from 60–70 Gy EBRT to 76–80 Gy EBRT was an appropriate treatment for localized prostate cancer. As BT has a higher conformality than EBRT, it has the potential to improve the therapeutic ratio by allowing higher doses to tumor. Three RCTs [12,13,14] and a systematic review have previously found that BT is associated with better biochemical control than either surgery or EBRT alone across all risk groups [15]. The improved biochemical control has allowed BT alone or EBRT plus BT to be included as standard treatment options by NCCN and The American Society of Clinical Oncology guidance documents for intermediate- to high-risk prostate cancer [1,7].

Advanced image-guided technology has shown that a low α/β ratio (for prostate cancer) concept enhanced the hypofractionated HDR-BT schedule [1,7,16]. This was undertaken because the short treatment period is convenient for patients, and the low α/β ratio implies the merit of improving biochemical control for prostate cancer while minimizing the toxicity around the normal tissues. In accordance with this notion, recent publications have reported good outcomes with acceptable toxicity [8,9,10,11,17].

In our previous works, we have employed HDR-BT monotherapy not only for low-risk patients but also for intermediate- and high-risk patients because it can provide an adequate dose distribution, even for extracapsular lesions and without EBRT [8,9,17]. Yang et al. reported that HDR-BT is clearly superior in the sparing of rectum, bladder, femoral heads, and normal tissue compared with IMRT [18]. Several other research groups have reported excellent outcomes, not only for low- and intermediate-risk but also for localized high-risk prostate cancer. Zamboglou et al. investigated HDR-BT monotherapy in 700 patients and obtained five-year biochemical control rates of 95% (100% in our data), 95% (95.6%), and 93% (90.4%) for their low-, intermediate-, and high-risk groups [10]. Thus, HDR brachytherapy is an option that has one of the highest curative potencies, not only for low- and intermediate-risk patients but also for high-risk patients. Our data demonstrate the potential of HDR-BT monotherapy to improve biochemical control in high-risk group compared to IG-IMRT and achieve equivalent outcomes for other risk groups. These data were obtained by direct comparison using the best statistical methods possible. The superiority of HDR-BT has been frequently reported in Western literature throughout low-, intermediate-, and high-risk groups, including three RCTs [12,13,14] and a systematic review [15,19]. Although its RCT was not done using the advanced IG-IMRT series, the Memorial group reported an improvement of a seven-year biochemical control rate through the addition of brachytherapy to the IMRT series (most cases received other treatment than IGRT). The Combo group (EBRT + LDR-BT or HDR-BT boost) group showed an improved biochemical control ratio of 81.4%, with high-dose IMRT (86.4 Gy/1.8 Gy fractionation) to 92% in the intermediate group [20]. Combo’s data are comparable to our seven-year biochemical control rate of 93.5% obtained with HDR-BT monotherapy; the 90.4% obtained with IG-IMRT with helical tomotherapy also seems not inferior to the Combo outcome. In the same way, it is interesting that HDR-BT monotherapy was found to be not superior to IG-IMRT with helical tomotherapy in other risk groups, even including the very-high-risk group. This is partly because of the high frequency of hormone use and the tendency for good outcomes in Asian populations, implying a racial difference. Tanaka et al. also reported that the five-year PSA control rates for the low-, intermediate-, high-, and very-high-risk groups were 95.7%, 91.4%, 91.4%, and 80.2%, respectively, in 1091 Japanese patients with helical tomotherapy [21].

The biochemical failure rates are markedly different among risk groups. They range from <5% in low-risk patients to 15% or more in high-risk patients [4]. Biochemical failure has been viewed as a poor surrogate for disease mortality for prostate cancer patients because it is closely linked to the need for salvage treatment that can impact quality of life and are related to progress to lethal diseases only for high- or very-high-risk risk patient. The influence of primary treatment on survival is often disguised by the use of hormonal therapy as there can be a long period of response, with a median time to resistant disease of seven years after radiotherapy [1,19]. Therefore, for patients with life expectancy less than 10 years, invasive procedure with morbidity will have limited value. From the current outcome, low- to intermediate-risk group could be good candidates to each of these treatments. In selected younger high-risk groups, patients would benefit from the knowledge that intensified treatments such as HDR-BT could improve PSA control, although no clear overall survival benefits have been proven.

In the toxicity analysis, the HDR-BT group showed a higher GU toxicity ratio than the IG-IGRT group, whereas there was an equivalent (or borderline significance) rate in GI toxicity. In the HDR group, higher biological equivalent dose correlated to higher GU toxicity. For example, the 49 Gy arm showed a higher five-year accumulated grade ≥ 2 genitourinary toxicity of 20.5% than those observed in the 45.5 Gy (2.4%) and 54 Gy arms (10.1%) [22]. In the IG-IMRT group, the initial 2.2 Gy/fractionation schedule begun with a wider planning target volume (PTV) margin, which resulted in 10% of GI cases having toxicity grades ≥2 [5]. When we reduced the fraction dose from 2.2 Gy to 2 Gy/fraction, excluding the rectal volume from PTV in calculating the optimization, the new schedule reduced GI toxicity to less than 2% [6], which concurred with data from a series of IG-IMRT studies from a pioneer group [2,3]. In general, BT may produce a higher incidence of GU toxicity than EBRT, including IG-IMRT, and a less incidence of GI toxicity [23].

Several limitations of this study should be mentioned. Firstly, it is a retrospective methodology that uses data from several institutions and deals with a rather small number of patients, which can cause an inherent bias. As treatment was selected at the discretion of the physicians at the time of consultation, the method of treatment was a nonrandomized variable. To confirm the reliability and potential of brachytherapy, studies with longer follow-ups with larger numbers of patients are needed before any concrete conclusion can be reached. The five-year biochemical outcome may not enough to estimate difference between treatments, especially when hormonal therapy is administered. Secondly, we did not examine several other potential factors that influence the PSA control rate. Our propensity score model could not replace a randomized controlled study because our model could only control known confounders; therefore, unknown confounders were not included. For example, in the United States, comorbidities that affect outcomes and toxicities are obesity and diabetes. Although these are not as frequently found in our population, they are epidemics in the US and in certain European countries [24]. Next, we could not examine the influence of technologic changes on HDR-BT. We thought that 3D planning techniques could improve the outcome than 2D planning. However, in the same time periods, dose fractionation was changed from 54 Gy/9 fractions to 45.5 Gy/7 fractions. Therefore, it is difficult to distinguish which factor affected the outcome. Finally, although our IG-IMRT schedule provided good outcomes [5,6], it is not a standard schedule nowadays, especially for intermediate- to high-risk patients [1,2,3,4]. Therefore, we escalated the prescribed dose to 78 Gy/39 fractions thereafter [5,6].

## 4. Materials and Methods

### 4.1. Patients

We included patients using the following eligibility criteria: (i) treatment with HDR-BT monotherapy or IG-IMRT for curative intent; (ii) clinical TNM stage T1–4 with histology-proven adenocarcinoma, N0, M0; (iii) available data on pretreatment prostate-specific antigen (initial PSA = iPSA) level, Gleason score sum (GS), and T classification; (iv) minimum one-year follow-up. Of the 646 patients, 21 were excluded because their follow-up was less than one year or because there was data missing.

Thus, the subjects of the study consisted of 623 patients with stage T1–T4 N0M0 prostate cancer. This included 353 patients treated using HDR-BT monotherapy (n = 353; treatment period: 1995–2013; 172 from Osaka University and 181 from Osaka National Hospital) and 270 patients treated with IG-IMRT with helical tomotherapy (n = 270; treatment period: 2007–2013; all patients from Ujitakeda Hospital). The patients’ characteristics are given in Table 1. All patients were staged according to the National Comprehensive Cancer Network (NCCN) risk classification as follows: (i) low: T1–T2a, GS 2–6, and iPSA <10 ng/mL; (ii) intermediate: T2b–T2c, GS 7, PSA 10–20 ng/mL; (iii) high: T3a, GS 8–10, PSA >20 ng/mL; and (iv) very high: T3b–T4 [1]. Phoenix definition (nadir, +2 ng/mL) or at the start of salvage hormonal therapy was used for definition of biochemical failure. Toxicity analysis was performed according to Common Terminology Criteria for Adverse Events version 4.0. All patients provided informed written consent. This study was conducted in accordance with the Declaration of Helsinki and permission of institutional review board (main institution; Kyoto Prefectural University of Medicine institutional review board permission, permission code; ERB-C-926) obtained at each institution where the study took place.

### 4.2. Treatment Planning

#### 4.2.1. Image-Guided Intensity-Modulated Radiotherapy

The detailed method of this study has been described elsewhere [5,6]. Briefly, we obtained computed tomography (CT) and magnetic resonance imaging (MRI) data for treatment planning approximately one week before treatment initiation. Each patient was instructed to empty the rectum and fill the bladder to reduce interfraction motion. CT examination was performed with a slice thickness of 2 mm in a supine position. We used CT images fused with MRI (T1w and T2w) to make meticulous radiotherapy planning. The clinical target volume (CTV) was defined for the prostate and proximal seminal vesicles or prostate only in the low-risk group (Damico’s classification: stage, T1c; Gleason score <7; and PSA <10 ng/mL). We started 2.2 Gy/fraction schedule, and the margin of expansion of the CTV and planning target volume was 5 mm in all directions, including the rectum. We used D95 (95% of PTV received at least the prescribed dose) of 74.8 Gy in 34 fractions (2.2 Gy/fraction) for intermediate- and high-risk patients. For the low-risk cases, 72.6 Gy in 33 fractions was used. We modified the prescribed dose, reduced to 74 Gy (D95) in 37 fractions for the high- and intermediate-risk groups and 72 Gy in 36 fractions for the low-risk group (2 Gy/fraction). The posterior margin (CTV–PTV expansion) was also reduced to 3 mm, with omitting rectal contour from PTV, excluding tumor that was located adjacent to the rectum. We used a 2.2 Gy fraction schedule between June 2007 and June 2009 and a modified 2 Gy fraction schedule from June 2009 to September 2013.

Organ at risks were set at the bladder and rectum as solid organs. We contoured rectal volumes from the rectosigmoid junction to the anal verge. Dose constraints set for the bladder and rectum were as follows: The rectal volume 35% and 18% received <40 Gy and <60 Gy, respectively, and bladder volume 50% and 25% received <40 Gy and <65 Gy, respectively.

#### 4.2.2. High-Dose-Rate Interstitial Brachytherapy Monotherapy

The method of applicator implantation has been described in detail elsewhere [8,9]. Initially, simple radiography-based treatment planning was used from 1995 to 2007 with the prescription dose point positioned 5 mm from one source in the central plane [2-dimensional (2D) planning]. Then, the method changed from 2D to 3-dimensional (3D) planning, and the patients were treated with 3D planning (i.e., CT-based planning) thereafter. For 3D setting, D90, D95 were used to evaluate if coverage of the planning target volume was adequate. CT-based planning, with or without MRI assistance, was performed using computer optimization (Nucletron, an Elekta Company, Veenendaal, The Netherlands, PLATO^®^ and Oncentra^®^ brachy, Elekta AB, Stockholm, Sweden), with or without manual modification. The major prescribed dose was 45.5 Gy per seven fractions, 54 Gy per nine fractions in 5 days, 49 Gy per seven fractions, and other. We began to implement HDR-BT monotherapy in the 1990s and employed a 54 Gy arm as an initial, frequently used schedule [8,9,12,13,14]. We changed this schedule from 9 fractions to 7 fractions to avoid treatment interruption due to holidays during Monday to Friday. Thereafter, the prescribed dose was changed to a 45.5 Gy arm and 49 Gy arm. A similar BED was maintained for the 49 Gy arm (163 Gy in α/β ratio = 3.0 Gy) for late-responding normal tissues, but BED (277 Gy in α/β ratio = 1.5 Gy) for cancer was elevated when compared with the initial 54 Gy arm (162 and 270 Gy), whereas the 45.5 Gy arm reduced both BED values (144 and 242 Gy; late toxicity and tumor). We aimed for a tumor BED of 240–270 Gy (α/β ratio = 1.5 Gy) and for a normal tissue BED of 144–162 Gy (α/β ratio = 3.0 Gy), with the difference expected to enhance therapeutic ratio [8,9,18]. The treatment machine used was the microSelectron-HDR^®^ (Nucletron an Elekta Company, Veenendaal, The Netherlands, Elekta AB, Stockholm, Sweden).

### 4.3. Statistical Analysis

R stat package [25] and StatView 5.0 statistical software were used for statistical analyses. R stat package was used only to calculate propensity score and IPTW. The chi-square test and Student’s *t*-test was used for percentage analysis. For skewed data, the Mann–Whitney U-test was used to compare means or medians. To analyze biochemical control rate, survival and accumulated toxicity, the Kaplan–Meier method, and the log-rank test was used. Univariate and multivariate analyses were performed by Cox’s proportional hazard model (all variables were included in the multivariate analysis). *p* < 0.05 was considered as statistically significant.

Unbalanced baseline characteristics could lead to selection bias, influencing the decision to undergo HDR-BT. The propensity score is defined here as the probability of being assigned to HDR-BT or IG-IMRT radiotherapy groups given the patients’ characteristics. The logistic regression model was used to calculate the propensity scores, considering the baseline covariates shown in Table 2 (age, T category, Gleason score, pretreatment PSA level, hormonal therapy; all variables were categorized variables). IPTW recalculated the treatment effects with a Cox model. Weighted survival analysis was performed using the IPTW method, i.e., patients who received HDR-BT were weighted by 1/propensity score, whereas patients who received IG-IMRT were weighted by 1/(1–propensity score).

## 5. Conclusions

HDR-BT as a monotherapy showed higher grade 2–3 genitourinary toxicity than IG-IMRT and similar gastrointestinal toxicity. HDR-BT and IG-IMRT showed equivalent outcome in low-, intermediate, and very-high-risk groups. For high-risk patients, HDR-BT showed potential to improve PSA control rate compared to IG-IMRT. Both IG-IMRT and HDR-BT were found to be acceptable treatment options for localized prostate cancer.

## Figures and Tables

**Table 1:** Characteristics and treatment factors of patients.

Variables	Strata	IG-IMRT			HDR-BT		*p*-Value
		n = 270			n = 353		
		No. or Median (range)	(%)		No. or Median (range)	(%)	
Age		71 (47–86)			71 (51–86)		**0.001**
T category	1	87	(32%)		95	(27%)	0.057
	2	118	(44%)		155	(44%)	
	3	64	(24%)		94	(27%)	
	4	1	(0.4%)		9	(3%)	
Pretreatment PSA	ng/mL	11.82 (1.971–658)			9.7 (4–265)		**0.002**
Gleason score	≤6	86	(32%)		117	(33%)	**0.0002**
	7	77	(29%)		147	(42%)	
	8≤	107	(40%)		89	(25%)	
NCCN risk classification	Low	48	(18%)		28	(8%)	**<0.0001**
	Intermediate	75	(28%)		145	(41%)	
	High	128	(47%)		141	(40%)	
	Very high	19	(7%)		39	(11%)	
Prescribed dose	74.8 Gy	102	(38%)	45.5 Gy	86	(24%)	NA
	72.6 Gy	24	(9%)	49 Gy	148	(42%)	
	74 Gy	119	(44%)	54 Gy	111	(31%)	
	72 Gy	25	(9%)	Others	8	(2%)	
	74.8 Gy	102	(38%)				
Hormonal therapy	Yes	176	(65%)		275	(78%)	**0.0005**
	No	94	(35%)		78	(22%)	
Follow-up	Months	74 (24–97)			84 (19–216)		**0.001**

Bold values indicate statistical significance. HDR-BT: high-dose-rate brachytherapy. IG-IMRT: image-guided intensity-modulated radiotherapy. NCCN: National Comprehensive Cancer Network. PSA: prostate-specific antigen.

**Table 2:** The five-year biochemical control rates between treatments.

Variable	Strata	PT No.	IG-IMRT	PT No.	HDR-BT	*p*-Value	
						Law Value	IPTW Correction
NCCN risk classification	Low	48	95.8%	28	100.0%	0.28	0.15
	Intermediate	75	92.0%	145	95.6%	0.42	0.6
	High	128	84.9%	141	90.4%	0.1	**0.04**
	Very high	19	87.1%	39	89.2%	0.38	0.6
	Total	270	92.9%	353	89.1%	0.18	0.07

Bold values indicate statistically significance. HDR-BT: high-dose-rate brachytherapy. IG-IMRT: image-guided intensity-modulated radiotherapy. NCCN: National Comprehensive Cancer Network.

**Table 3:** Univariate and multivariate analysis for biochemical control rate using Cox proportional hazards model.

Variable	Strata	Biochemical Control					
		Univariate Analysis			Multivariate Analysis		
		HR	95% CI	*p*-Value	HR	95% CI	*p*-Value
Age, years	<72	1	(referent)	-	1	(referent)	-
	72≤	0.62	0.38–1.02	0.06	0.64	0.39–1.07	0.09
T category	T1–2	1	(referent)	-	1.00	(referent)	-
	T3–4	2.36	1.49–3.75	**0.0003**	1.97	1.08–3.49	**0.02**
Gleason score	≤7	1	(referent)	-	1.00	(referent)	-
	8≤	2.36	1.49–3.75	**0.0003**	1.97	1.08–3.49	**0.02**
Pretreatment PSA (ng/mL)	<20	1	(referent)	-	1.00	(referent)	-
	20≤	2.78	1.75–4.41	**<0.0001**	2.53	1.44–4.44	**0.001**
NCCN risk classification	Low–Intermediate	1	(referent)	-	NA		
	High–Very high	2.85	1.67–4.87	**0.0001**			
Hormonal therapy	No	1	(referent)	-	1.00	(referent)	-
	Yes	0.95	0.56–1.59	0.84	1.88	0.98–3.57	0.054
Treatment modality	HDR-BT	1	(referent)	-	1.00	(referent)	-
	IG-IMRT	1.38	0.85–2.24	0.18	0.65	0.39–1.08	0.10

Bold values indicate statistical significance. CI = confidence interval; HR = hazard ratio, NA = not available.

**Table 4:** Late toxicities according to modalities.

Toxicities	Strata	IG-IMRT		HDR-BT		*p*-Value
		n = 270		n = 353		
		No.	(%)	No.	(%)	
Gastrointestinal	0	224	(83%)	310	(87%)	0.094
	1	30	(11%)	33	(9%)	
	2	11	(4%)	10	(3%)	
	3	5	(2%)	1	(0.3%)	
Genitourinary	0	222	(82%)	186	(52%)	**<0.0001**
	1	36	(13%)	100	(28%)	
	2	11	(4%)	57	(16%)	
	3	1	(0%)	10	(3%)	

Bold values indicate statistical significance. IG-IMRT: image guided intensity modulated radiotherapy. HDR-BT: high-dose-rate brachytherapy.

**Table 5:** Univariate and multivariate analysis for genitourinary toxicity grade 2 or more.

Variable	Strata	Genitourinary Toxicity Grade ≥ 2					
		Univariate Analysis			Multivariate Analysis		
		HR	95% CI	*p*-Value	HR	95% CI	*p*-Value
Age, years		1.004	0.97–1.03	0.80	1.016	0.98–1.05	0.36
NCCN risk classification	Low–Intermediate	1	(referent)		1	(referent)	
	High–Very high	0.86	0.55–1.34	0.51	0.925	0.57–1.43	0.75
Hormonal therapy	No	1	(referent)	-	1	(referent)	
	Yes	1.055	062–1.77	0.83	0.89	0.50–1.57	0.69
Treatment modality	HDR-BT	1	(referent)		1	(referent)	
	IG-IMRT	3.747	2.00–7.00	**<0.0001**	3.91	2.07–7.39	**<0.0001**

Bold values indicate statistically significance. CI = confidence interval; HR = hazard ratio.
